# Beyond the sugar tax: public acceptability of a comprehensive food policy bundle in urban Ghana

**DOI:** 10.3389/fnut.2026.1847802

**Published:** 2026-08-25

**Authors:** Kasim Abdulai, Emil Kafui Amenu, Portia Bangkuu, Ivan Addae-Mensah, Francis Adane, Abass Daudi

**Affiliations:** 1 Translational Nutrition Research Group, Department of Nutrition and Dietetics, University of Cape Coast, Cape Coast, Ghana; 2 Department of Population and Health, Faculty of Social Sciences, University of Cape Coast, Cape Coast, Ghana; 3 Department of Health Policy, Planning and Management, School of Public Health, University of Ghana, Accra, Ghana; 4 Tamale Teaching Hospital, Tamale, Ghana

**Keywords:** food marketing restrictions, front-of-pack warning labeling, Ghana, non-communicable diseases, public support, sugar-sweetened beverage tax, Theory of Planned Behavior

## Abstract

**Background:**

The escalating burden of diet-related non-communicable diseases in Ghana has prompted the government to implement a 20% excise tax on sugar-sweetened beverages through the Excise Duty (Amendment) Act 1108 of 2023. While fiscal measures have garnered significant advocacy, public health experts emphasize the need for a “double-duty” policy bundle, including food marketing restrictions, front-of-pack warning labeling, and public food procurement policies. The problem is that public support for this broader bundle remains understudied in urban Ghana, risking implementation failure.

**Objective:**

This study evaluated the prevalence and socio-political determinants of public support for a comprehensive suite of food environment policies among adults in the Cape Coast Metropolis, Ghana, and explored underlying motivations.

**Methods:**

Convergent mixed-methods design. Cross-sectional survey of 450 adults (18–69 years) using multi-stage cluster sampling. Support measured via validated 5-point Likert scales. Twenty-four in-depth interviews provided qualitative depth.

**Results:**

Support was highest for front-of-pack warning labeling (81.4%), followed by marketing restrictions (76.8%), the sugar-sweetened beverage tax (71.2%), and public procurement (65.6%). Perceived policy effectiveness (adjusted odds ratio = 3.45) and trust in the Ghana Health Service (adjusted odds ratio = 1.92) were the strongest predictors. Qualitative findings revealed that support for the tax increases significantly if revenues are ring-fenced for the National Health Insurance Scheme.

**Conclusion:**

Policymakers should immediately prioritize front-of-pack warning labeling and marketing restrictions as highly acceptable “empowerment” policies, while simultaneously legislating transparent earmarking of sugar-sweetened beverage tax revenues for non-communicable disease treatment and prevention. Future research should test these policies post-implementation using longitudinal designs.

## Introduction

The global health landscape is currently defined by an alarming shift in the burden of disease, with non-communicable diseases (NCDs) now responsible for approximately 41 million deaths annually (1). A staggering 85% of these premature deaths occur in low- and middle-income countries (LMICs), where health systems are often ill-equipped to manage chronic, lifelong conditions (1). In Ghana, the situation has reached a critical threshold; NCDs primarily cardiovascular diseases, cancers, chronic respiratory diseases, and diabetes account for approximately 45% of all national deaths (1). This escalating crisis is fuelled by a rapid epidemiological transition characterized by urbanization, sedentary lifestyles, and a deteriorating food environment (2). Specifically, no previous study in Ghana has quantitatively examined public support for front-of-pack warning labels and food marketing restrictions alongside the sugar-sweetened beverage tax using a mixed-methods design, leaving a critical evidence gap for integrated policy design.

The Ghanaian food environment is increasingly dominated by ultra-processed foods (UPFs) and sugar-sweetened beverages (SSBs), which are heavily marketed and widely accessible even in low-income urban areas (3). Examples of such products commonly consumed in Cape Coast include foreign-branded carbonated drinks (Coca-Cola, Fanta, Sprite), locally produced fruit juices (e.g., FanChoco, FanIce, and bottled sachet drinks like “Supermal”), and ultra-processed snacks (biscuits, instant noodles, and meat pies) sold widely at Kotokuraba market and school canteens. The 2023 STEPS report reveals a concerning dietary profile: 76% of Ghanaians do not consume the recommended five servings of fruits and vegetables daily, while salt intake is pervasive, with over 90% of adults adding salt during meal preparation (1). These behaviors contribute to a rising prevalence of overweight and obesity, which has nearly tripled among certain demographics over the past three decades (4). In urban centers like the Cape Coast Metropolis, studies have found obesity rates as high as 39% among market women, directly linked to poor dietary diversity and the frequent consumption of high-calorie street foods (5).

Recognizing the need for urgent intervention, the Government of Ghana enacted the Excise Duty (Amendment) Act 1108 in 2023, imposing a 20% excise tax on the ex-factory price of SSBs, including fruit juices and carbonated drinks (6). The Act specifically applies to all non-alcoholic, ready-to-drink beverages with added sugar or other sweeteners, including carbonated soft drinks (e.g., Coca-Cola, Sprite, Fanta), fruit juices with added sugar (e.g., Chivita, FanIce), energy drinks, and sweetened bottled water, irrespective of whether they are locally produced or imported. This legislation was the result of vigorous advocacy by a coalition of academics and civil society organizations (CSOs), including the “Healthier Diets for Healthy Lives” (HD4HL) project led by the University of Ghana School of Public Health (6). However, while the SSB tax is a landmark fiscal policy, global best practices suggest that it must be part of a broader “double-duty” policy bundle to effectively transform the food system (7). This bundle includes Food Marketing Restrictions Policy (FMRP) to protect children, Front-of-Pack Warning Labeling (FOPWL) to empower consumers, and Public Food Procurement Policy (PFPP) to ensure healthy food availability in state institutions like schools and hospitals (7).

Public support is the cornerstone of successful policy implementation. Without it, interventions risk being framed as “nanny-state” overreaches, making them vulnerable to industry interference and political reversal (8). In Ghana, industry actors have already argued that the public perceives these taxes as an additional economic burden during times of inflation (8). Conversely, advocacy groups suggest that Ghanaians are generally supportive of health measures if the rationale is clear and the revenue is used transparently (9). A recent nationwide poll indicated that 68% of residents supported government efforts to tax harmful foods, but this support jumped to nearly 80% if revenues were “ring-fenced” for health interventions (9).

Despite these national trends, there is a significant gap in localized, evidence-based research that examines support for the full suite of food environment policies. Most studies have focused almost exclusively on the SSB tax, neglecting the potential for high public acceptability of marketing and labeling regulations (7). Furthermore, the specific urban dynamics of the Cape Coast Metropolis a historic city characterized by high educational levels but also significant food insecurity in its informal sectors provide a unique backdrop for examining how socio-economic status and institutional trust influence policy support (10).

This study addresses this knowledge gap by employing a mixed-methods design to investigate public attitudes toward the SSB tax, FMRP, FOPWL, and PFPP in Cape Coast. The research aims to quantify the prevalence of support, identify the socio-demographic and psychological predictors of this support, and explore the qualitative reasons why some policies may be more palatable to the Ghanaian public than others. By situating the study within the Cape Coast Metropolis, the research offers granular insights into how urban Ghanaians navigate their changing food environment and what they expect from government health interventions (5).

To comprehensively analyze the determinants of public support for nutrition policies, this study integrates the Social Ecological Model (SEM) and the Theory of Planned Behavior (TPB). These frameworks allow for a multi-level analysis that accounts for both individual psychological factors and broader environmental and institutional influences (11, 12).

The theoretical framework of this study integrates the Social Ecological Model (SEM) and the Theory of Planned Behavior (TPB) to provide a comprehensive understanding of how individuals are embedded within a hierarchy of social systems that shape their health perceptions and policy support. Within the specific context of the Cape Coast Metropolis, the SEM accounts for individual-level factors such as personal sugar-sweetened beverage (SSB) consumption, age, and education level that directly influence a respondent’s “readiness” to support fiscal or labeling interventions (1). This extends to the interpersonal level, where social norms and family structures particularly the role of mothers as food gatekeepers shape attitudes toward appropriate marketing practices for children (11–13). Furthermore, the community and organizational landscape, including the physical proximity to the Kotokuraba market and the prevalence of fast-food vendors around the University of Cape Coast, provides the necessary geographic context for policy relevance, while the macro-political level involves how trust in government institutions and the 2023 Excise Duty Act influence the perceived legitimacy of state intervention (10). While the SEM establishes this environmental context, the TPB provides the predictive mechanism for individual support by determining how intentions are formed through attitudes toward a policy’s benefits, subjective norms from influential referent groups, and perceived behavioral control or “political efficacy” (13–15). The integration of these theories is particularly robust in the Ghanaian context, as the SEM captures the “causes of the causes,” such as urbanization and market dynamics, while the TPB identifies the specific cognitive filters, like perceived fairness and political trust, that mediate between the environment and policy acceptability (16). Consequently, while a high-income resident may support an SSB tax due to the perceived “control” to purchase healthier alternatives (TPB), a market woman might favor marketing restrictions based on her observation of the community-level influence of aggressive advertising on her children (SEM) (13, 14).

## Literature review

### The global and Ghanaian food environment

The global nutrition transition has led to increased consumption of ultra-processed foods and sugar-sweetened beverages, particularly in low- and middle-income countries undergoing rapid urbanization (1, 2). In Ghana, the 2023 STEPS report documented that 76% of adults do not meet the recommended daily intake of fruits and vegetables, while over 90% add salt during meal preparation (1). These dietary patterns are driven by multiple factors: the proliferation of fast-food vendors, aggressive marketing by transnational food corporations, and the low cost of highly processed relative to fresh foods (3, 4).

### Empirical evidence on public acceptability of food policies

International evidence shows that public support for food environment policies varies by policy type. A systematic review and meta-analysis by Eykelenboom et al. (17) found that front-of-pack labeling consistently receives the highest support (often >80%), followed by marketing restrictions, while sugar-sweetened beverage taxes receive moderate support (50–70%) that increases when revenues are earmarked for health. A systematic review of evidence from the African context similarly found that SSB taxes have generated increased revenue in some countries, though their long-term sustainability and public acceptability remain mixed (13). In South Africa, Bosire et al. (18) reported that support for a sugar-sweetened beverage tax increased from 49 to 71% when respondents were told the revenue would fund health programs. In Mexico, warning labels were framed by consumers as empowering tools rather than coercive regulations (19).

In Ghana, recent studies have focused predominantly on the sugar-sweetened beverage tax. Singh et al. (20) found that stakeholder support for health taxes was strong but conditional on transparent revenue use. Smith et al. (8) documented that industry actors argue the public perceives taxes as an economic burden, while advocates suggest high support if revenues are ring-fenced. A South African study further demonstrated that an effective and sustainable sugar tax is contingent on public awareness of its purpose and overall acceptance, reinforcing the critical role of public support (14). However, no published study in Ghana has simultaneously quantified public support for front-of-pack warning labels, marketing restrictions, and public procurement policies using a mixed-methods approach. This gap is critical because the World Health Organization recommends a “double-duty” bundle of policies for effective food system transformation (7).

### Theoretical framework: integration of the social ecological model and Theory of Planned Behavior

This study integrates the Social Ecological Model (SEM) and the Theory of Planned Behavior (TPB). The SEM identifies multiple levels of influence on health behavior: individual (e.g., age, education, sugar-sweetened beverage consumption), interpersonal (e.g., family norms around food), community (e.g., proximity to markets), and macro-policy (e.g., trust in government) (11, 12). The TPB posits that intention to support a policy is shaped by attitude (perceived benefits), subjective norm (perceived social pressure), and perceived behavioral control (e.g., political efficacy) (16).

These theories harmonize by recognizing that macro-level factors (e.g., urban food environment) shape individual cognitive predictors. For example, a market woman in Cape Coast may oppose a tax (low attitude) because she perceives it as unaffordable, but support marketing restrictions (high attitude) because she observes aggressive advertising targeting her children – a community-level influence captured by SEM and mediated by TPB constructs. This integrated framework guided our variable selection and mixed-methods design.

## Methods

### Study design

This study utilized a convergent mixed-methods design, characterized by the simultaneous collection and independent analysis of quantitative and qualitative data, followed by a merged integration phase (15). The rationale for this design is “complementarity”; quantitative data provide a statistically representative measure of support across the metropolis, while qualitative data provide the “lived experience” and “contextual nuances” that survey instruments often miss (16). Specifically, the quantitative strand measures prevalence and statistical predictors, while the qualitative strand explains why certain policies (e.g., front-of-pack labeling) are more acceptable than others (e.g., taxation). This design is essential for policy research because high statistical support without understanding underlying concerns (e.g., distrust of revenue management) can lead to implementation failure. This approach is particularly robust for policy research, as it allows researchers to identify contradictions such as high support for a tax despite deep distrust in government revenue management (8).

### Study setting

Cape Coast, traditionally known as “Oguaa” (meaning “market”), is the capital of the Central Region of Ghana (21). It is an ideal setting for food policy research due to its rapid urbanization and high density of educational institutions. As of 2021, the metropolis has a population of 189,925, with a significant proportion of the workforce engaged in trading, artisanship, and fishing. The city’s food environment is shaped by high-paced urban routines, leading to a reliance on prepared and pre-packaged foods from the Kotokuraba and Abura markets. Furthermore, Cape Coast is often described as water-scarce, which increases the consumption of bottled and sachet drinks, many of which fall under the SSB category.

### Sampling and study population

The target population included adults aged 18 to 69 who had resided in the Cape Coast Metropolis for at least 6 months. The sample size of 450 was calculated using the Cochran formula for cross-sectional studies: *n* = (*Z*^2^ × *p* × (1−*p*))/e^2^, assuming a 50% expected prevalence of policy support (*p* = 0.50) to maximize sample size, a 95% confidence level (*Z* = 1.96), and a margin of error of 5% (e = 0.05). This yielded a minimum of 384 participants, inflated to 450 to account for 15% non-response or incomplete data.

To enhance representativeness, the total sample was proportionally allocated across selected electoral areas according to estimated population size. Within sampled households, eligibility was assessed before selection of one respondent using the Kish grid. Data collection took place between 15 January 2024 and 30 March 2024. Where more than one eligible respondent was present, only one was interviewed. Households in which no eligible respondent was available after two revisits were treated as non-responding and replaced using the next eligible household within the sampling interval. A purposive subsample of 24 participants was selected from the quantitative survey respondents for in-depth interviews (IDIs). Selection was stratified by age, sex, and “policy stance” (Supporter, Neutral, or Opponent) to ensure maximum variation in perspectives. The sampling frame for probability proportional to size (PPS) was the 2021 Ghana Population and Housing Census data for the Cape Coast Metropolis, obtained from the Ghana Statistical Service. The population of each electoral area was used to determine the proportional allocation of participants. Within each selected electoral area, the specific communities (listed in Table 1) were randomly selected from the census enumeration areas using a computer-generated random number sequence. Households within each community were selected using systematic sampling with a fixed interval (every 5th household) after a random starting point. Within each selected household, one eligible adult (aged 18–69 years) was selected using a Kish grid to ensure random selection when multiple eligible adults were present. Data collection took place between 15 January 2024 and 30 March 2024. The overall response rate was 92.3% (450 completed interviews out of 487 eligible households approached). Non-response was primarily due to household members being absent after two revisits (*n* = 37, 7.6%). To account for the complex survey design (stratification by electoral area, clustering by community, and weighting by population size), all analyses were conducted using survey-weighted procedures (Stata’s svy: commands) to obtain correct standard errors and population-representative estimates.

**Table 1:** Electoral areas, selected communities, and number of participants.

Electoral area	Communities selected	Sampling method	Number of participants (*n*)
Cape Coast North	Abura, Nkanfoa	Probability proportional to size	112
Cape Coast South	Kotokuraba, OLA Estates	Probability proportional to size	98
Bakano	Bakano New Site, Amamoma	Probability proportional to size	85
Pedu	Pedu Junction, Bakaano Extension	Probability proportional to size	80
Anafo	Anafo Central, Duakor	Probability proportional to size	75
Total	10 communities		450

Households were selected using systematic sampling (every 5th household). Within each household, one eligible adult was selected using a Kish grid.

### Instruments

The KoboToolbox was employed for data collection. The questionnaire was developed from previously used items on public attitudes toward health taxes and food policy measures and adapted to the Ghanaian context. Items measuring political trust, perceived fairness, and perceived effectiveness were reviewed for contextual relevance and pretested before field deployment. Internal consistency of multi-item constructs was assessed using Cronbach’s alpha, with acceptable reliability defined *a priori* as *α* ≥ 0.70. The full version of the quantitative questionnaire, including all sections and items, is provided as Supplementary File 1. The main manuscript contains an abridged version for readability, with the complete instrument available in the Supplementary materials.

### Data collection procedures

Informed consent was first obtained from the study participants. Trained research assistants collected data through face-to-face interviews to ensure rigorous data quality, research assistants participated in 3 days of intensive training, and a pre-test was conducted in Elmina to refine the survey items before full deployment. Complementing this, qualitative data were gathered through in-depth interviews (IDIs) with a purposive subsample of 24 participants. These interviews were conducted in the participants’ preferred language either Fante or English to ensure a nuanced understanding of their attitudes. All qualitative sessions were audio-recorded and subsequently transcribed verbatim to maintain the integrity of the participants’ perspectives for analysis.

### Data analysis

Quantitative data were analyzed using Stata version 17.0. For quantitative analysis, responses to each policy item were first summarized descriptively. For regression analysis, an overall binary outcome variable, “support for the policy bundle,” was created and coded as 1 for respondents who supported or strongly supported all four policies and 0 otherwise. Independent variables considered included age, sex, education, SSB consumption frequency, NCD awareness, political trust, perceived fairness, perceived effectiveness, and perceived tax burden. Variables associated with the outcome at *p* < 0.20 in bivariate analysis, together with theoretically relevant covariates, were entered into the multivariable logistic regression model. The logistic regression model was specified as: logit(P(Y = 1)) = *β*₀ + β₁(Education_tertiary) + β₂(NCD_awareness_high) + β₃(Trust_GHS_high) + β₄(Perceived_effectiveness_high) + β₅(Perceived_burden_high) + β₆(SSB_daily) + *ε*. where P(Y = 1) is the probability of supporting all four policies, β₀ is the intercept, β₁–β₆ are log-odds coefficients, and ε is the error term. Multicollinearity was assessed using variance inflation factors (VIF). All VIF values were below 2.5 (mean VIF = 1.38), indicating no problematic collinearity. Model fit was evaluated using the Hosmer–Lemeshow goodness-of-fit test (χ^2^ = 8.32, *p* = 0.40), indicating adequate fit. The area under the receiver operating characteristic curve was 0.81 (95% CI: 0.77–0.85), demonstrating good discriminative ability.

Adjusted odds ratios (AORs) with 95% confidence intervals were reported. Multicollinearity was assessed using variance inflation factors, and model fit was examined using appropriate goodness-of-fit procedures. Qualitative data from the in-depth interviews were analyzed using a thematic approach to provide a deeper understanding of the motivations behind policy acceptability. The process began with the verbatim transcription of audio recordings, which were then imported into NVivo software for systematic management and coding. An inductive-deductive coding strategy was applied, where initial codes were derived from the research objectives and the Theory of Planned Behavior (TPB) framework, while emerging themes were identified through an iterative reading of the transcripts. Thematic saturation was assessed prospectively during data collection. After each in-depth interview, the research team met to review transcripts and identify new codes. No new themes emerged after the 20th interview; the remaining four interviews (17, 18, 20, 22) confirmed that saturation had been reached. Data collection therefore stopped at 24 interviews based on this emergent criterion, not a predetermined target. In accordance with the convergent mixed-methods design, the quantitative and qualitative findings were integrated during the interpretation phase. Two members of the research team independently reviewed a subset of transcripts to refine the coding framework and improve analytic consistency. Coding discrepancies were discussed and resolved through consensus. Illustrative quotations were selected to reflect both dominant and divergent views across participants.

#### Positionality statement

The lead author (KA) is a male nutrition researcher with professional experience in public health advocacy for SSB taxation. This background may have introduced a bias toward viewing taxation favorably. To mitigate this, a reflexive journal was maintained throughout analysis, and a second coder (PB, female, with no prior involvement in tax advocacy) independently coded 30% of transcripts. Disagreements were resolved through consensus. Participants were informed that the researcher was from a university, not a government agency, to reduce social desirability effects.

### Ethical considerations

Ethical clearance was obtained from the University of Cape Coast Institutional Review Board The approval letter was issued on 14th November 2023 and carried Ethical Clearance ID UCCIRB/CHAS/2023/172 and institutional reference number IRB/C3/Vol.1/0486. Permission was sought from community leaders before data collection commenced. The study’s purpose, procedures, risks, and benefits were explained to all participants in their local language. Both verbal and written informed consent were obtained from all participants. Participation was entirely voluntary, and participants were assured of their right to withdraw at any time without consequence. Confidentiality was maintained by using unique identification codes for all questionnaires and data files. The complete data collection instruments (questionnaire and in-depth interview guide) are provided in Appendix B.

## Results

No missing data were present for the primary outcome variables. Normality of continuous composite scores was assessed via Shapiro–Wilk test (*p* > 0.05 for all), permitting parametric summaries. Internal consistency for multi-item scales is reported in Table 2.

**Table 2:** Variable definitions, measurement units, and sources.

Variable	Definition	Unit/scale	Source/adaptation
Support for SSB tax	Agreement with 20% excise tax on sugar-sweetened beverages	5-point Likert (1 = Strongly oppose to 5 = Strongly support)	Adapted from Eykelenboom et al. (2019) (17)
Support for FOPWL	Agreement with mandatory warning labels on front of pack	5-point Likert (same)	Adapted from Konlan et al. (2023) (19)
Support for FMRP	Agreement with restricting marketing of unhealthy foods to children	5-point Likert (same)	Adapted from Tandoh et al. (2023) (24)
Support for PFPP	Agreement with healthy public food procurement in institutions	5-point Likert (same)	Adapted from Nanema et al. (2025) (7)
Perceived effectiveness	Belief that the policy bundle will reduce NCDs	5-point Likert (summed, then dichotomised: high/low at median)	Smith et al. (2025) (8)
Trust in Ghana Health service	Confidence that GHS will implement policies fairly	5-point Likert (summed, dichotomised)	Singh et al. (2023) (20)
Perceived tax burden	Extent to which SSB tax is seen as financially burdensome	5-point Likert (summed, dichotomised)	Eykelenboom et al. (2019) (17)
NCD awareness	Knowledge of NCD causes and consequences	10-item quiz (score ≥7 = high)	Ghana STEPS Report (1)
SSB consumption frequency	Self-reported intake of sugar-sweetened beverages	Daily/2–5 times per week/Once a week or less	Apprey et al. (2023) (5)
Education	Highest level of formal education	No formal/Primary/Secondary/Tertiary	Standard demographic
Age	Completed years	18–29/30–49/50–69	Standard demographic
Sex	Biological sex	Male/Female	Standard demographic

Cronbach’s alpha for multi-item constructs: perceived effectiveness (*α* = 0.84), trust in GHS (*α* = 0.79), perceived tax burden (*α* = 0.81).

### Socio-demographic and behavioral profile

The study analyzed 450 participants (Table 1). The sample was slightly more female (54.2%), reflecting the demographic distribution of Cape Coast. A significant proportion of the sample (42.4%) reported consuming SSBs multiple times per week, consistent with the high availability of these products in urban markets (1).

### Prevalence of policy support

The data demonstrate that public support for food policies in Cape Coast is high across the board, with FOPWL and FMRP receiving more enthusiastic approval than the SSB tax. As shown in Table 2, FOPWL is the most popular intervention (81.4% total support), followed by FMRP (76.8%). This suggests that “information-based” and “protection-based” policies are more acceptable to the urban Ghanaian public than “price-based” interventions like the SSB tax (71.2%).

### Multivariable predictors of support

In the adjusted model (Table 3), respondents with tertiary education had higher odds of supporting the full policy bundle than those with lower educational attainment (AOR = 1.85, 95% CI: 1.32–2.58). High NCD awareness (AOR = 2.10, 95% CI: 1.54–2.88), high trust in the Ghana Health Service (AOR = 1.92, 95% CI: 1.40–2.65), and high perceived policy effectiveness (AOR = 3.45, 95% CI: 2.50–4.78) were positively associated with support. In contrast, respondents reporting a high perceived tax burden (AOR = 0.62, 95% CI: 0.45–0.85) and those consuming SSBs daily (AOR = 0.48, 95% CI: 0.32–0.71) had lower odds of supporting the entire policy package.

**Table 3:** Socio-demographic and behavioral characteristics of participants.

Characteristic	Frequency (*n* = 450)	Percentage (%)
Sex		
Male	206	45.8
Female	244	54.2
Age group		
18–29	158	35.1
30–49	212	47.1
50–69	80	17.8
Education		
No Formal/Primary	98	21.8
Secondary	194	43.1
Tertiary	158	35.1
SSB consumption		
Daily	72	16.0
2–5 times a week	191	42.4
Once a week or less	187	41.6
NCD awareness		
High	265	58.9
Low	185	41.1

### Mixed-methods integration and qualitative themes

Overall, the integrated findings demonstrated substantial convergence between the quantitative and qualitative strands. Policies with the highest quantitative support, particularly FOPWL and FMRP, were also the ones most consistently framed in interviews as fair, informative, and protective. By contrast, support for the SSB tax was more conditional and context-dependent in both strands, with participants expressing willingness to endorse taxation when its health rationale and revenue use were clearly communicated. PFPP received comparatively lower support quantitatively, and qualitative accounts suggested that this was driven less by opposition to the policy objective itself than by skepticism regarding implementation capacity and accountability within public institutions. The qualitative IDIs (*n* = 24) identified three central themes that explain the quantitative trends (Table 4).

**Table 4:** Levels of support for the four food policies in Cape Coast.

Policy intervention	Strongly support (%)	Support (%)	Neutral (%)	Oppose (%)	Strongly oppose (%)
SSB tax	32.4	38.8	11.2	12.6	5.0
FMRP	38.2	38.6	10.4	10.0	2.8
FOPWL	45.1	36.3	9.2	7.0	2.4
PFPP	28.4	37.2	22.1	8.8	3.5

#### Theme 1: empowerment vs. coercion

Participants preferred FOPWL and FMRP because these were seen as “empowering” the consumer to make a choice rather than “forcing” them through prices.

“The label is good because it tells me the truth. If I see it says ‘High Sugar,’ I can decide if I want to die early or live for my children. But the tax, sometimes it just makes us poorer without changing our heart” (Quote, IDI, Female, 42, Market Trader).

#### Theme 2: the “moral shield” for children

Support for FMRP was driven by a collective desire to protect children from the “tricks” of advertisements.

“These advertisements use celebrities our children love. It is not fair. A child cannot tell what is healthy. The government must act as a shield for them” (Quote, IDI, Male, 38, Teacher).

#### Theme 3: the “ring-fencing” requirement

Support for the SSB tax is highly conditional on transparency. Many participants expressed “tax fatigue” but were willing to pay if the revenue directly funded the NHIS.

“We pay so many taxes in Ghana. If this sugar tax goes into the general pot, I am against it. But if they say it will buy insulin for the clinics in Cape Coast, then it is a blessing” (Quote, IDI, Male, 55, Retiree).

## Discussion

This mixed-methods study adds to emerging evidence on public acceptability of food environment policies in Ghana by showing that support extends beyond SSB taxation to include warning labels, marketing restrictions, and public food procurement measures. The findings reveal high overall support for all four interventions FOPWL (81.4%), FMRP (76.8%), SSB tax (71.2%), and PFPP (65.6%) with perceived effectiveness emerging as the strongest predictor of support (AOR: 3.45). The qualitative data illuminate the contextual nuances underlying these patterns, revealing that Ghanaians frame labeling and marketing restrictions as “empowering” measures that preserve autonomy, while viewing taxation as potentially “coercive” unless revenues are transparently earmarked for health. These findings carry significant implications for policy design and implementation in Ghana and similar LMIC contexts undergoing rapid nutrition transition (Table 5).

**Table 5:** Multivariable predictors of overall policy bundle support.

Predictor variable	Adjusted odds ratio (AOR)	95% confidence interval (CI)	*p*-value
Tertiary education	1.85	[1.32–2.58]	0.002
High NCD awareness	2.10	[1.54–2.88]	<0.001
High trust in GHS	1.92	[1.40–2.65]	<0.001
High perceived effectiveness	3.45	[2.50–4.78]	<0.001
High perceived tax burden	0.62	[0.45–0.85]	0.003
Daily SSB consumer	0.48	[0.32–0.71]	<0.001

The high support for FOPWL can be interpreted through the Theory of Planned Behavior’s construct of “Attitude.” Participants evaluated labels as highly beneficial because they provide the informational foundation for behavioral control (23). In contrast to the SSB tax, which can be perceived as an “external” constraint, labels are viewed as “internal” empowerment tools (19). The Social Ecological Model helps explain the robust support for FMRP. In Ghanaian society, where communal child-rearing is a core “interpersonal” and “community” value, aggressive marketing of unhealthy foods to children is seen as an affront to the social fabric. This moral mandate for child protection overrides concerns about corporate freedom, explaining why FMRP support (76.8%) is higher than SSB tax support (71.2%) (24). The observed hierarchy of support with FOPWL and FMRP outperforming SSB tax and PFPP aligns with the Social Ecological Model’s emphasis on how macro-level policy characteristics interact with community-level norms and individual-level autonomy concerns. At the policy level, FOPWL and FMRP are perceived as “enabling” interventions that expand consumer choice and protect vulnerable populations (children) without directly constraining purchasing power (12, 24). This finding mirrors evidence from Mexico, where front-of-package warning labels were associated with self-reported behavior change and were framed by consumers as tools for informed choice rather than coercive regulation (18). In contrast, the SSB tax operates at the economic level of the SEM, directly affecting household budgets in a context where 42.4% of the sample consumes SSBs multiple times weekly and where inflation remains a salient political concern (19).

The qualitative theme of “empowerment versus coercion” reflects a deeper socio-cultural preference in Cape Coast for policies that respect individual agency while providing the informational infrastructure for healthier choices. This preference is consistent with Ghanaian stakeholder views documented by Singh et al., who found that health taxes were often perceived primarily as revenue instruments rather than consumption-reduction measures, and that public framing emphasizing health benefits and local evidence could strengthen acceptability (20).

This finding resonates with evidence from South Africa, where analysis of packaged foods revealed widespread child-directed marketing and supported regulatory actions to protect children from unhealthy product promotion (22). In the Ghanaian context, where extended family networks and communal child-rearing practices are normative, the framing of FMRP as a “moral obligation” rather than merely a health measure may explain its high acceptability across socio-demographic groups (Table 6).

**Table 6:** Joint display of integrated mixed-methods findings.

Policy	Quant support	Qual interpretation	Meta-inference
SSB tax	71.2%	Concerns about “money for government” vs. “money for health.”	Support is fragile and depends on visible health spending (Earmarking).
FOPWL	81.4%	High value placed on transparency and “the truth.”	Labeling is seen as a “Common Sense” policy with minimal political friction.
FMRP	76.8%	Protective instincts toward children and social stability.	Restricting marketing is viewed as a moral obligation, not just a health one.

Our finding that support for the SSB tax (71.2%) is lower than for labels mirrors trends in other LMICs and developed economies (17). In South Africa and Mexico, initial tax support was tempered by concerns about economic regressivity, yet support for “earmarked” taxes remained high (13). A systematic review and meta-analysis found that public support for a sugar-sweetened beverage tax increased by approximately 30 percentage points when revenues were hypothetically earmarked for health promotion programs, a pattern observed across multiple countries including those in Africa (17). The current study confirms this in Cape Coast; participants are not “anti-tax” but rather “pro-accountability” (9).

The high support for FOPWL (81.4%) aligns with recent evidence from the “Healthier Diets for Healthy Lives” project, which suggests that Ghanaians have a high “objective understanding” of simple labels even when their general nutrition knowledge is low (19). This confirms that labels are a “Best Buy” not just because of their clinical effectiveness, but because of their extreme public acceptability (4). However, it is important to note that our measure of institutional trust focused on the Ghana Health Service (GHS), a health delivery agency. The qualitative findings (Theme 3) revealed that participants’ primary distrust was fiscal, concerns about government revenue management, not about health professionals. Trust in the GHS may therefore not fully capture the “fiscal distrust” that undermines support for the SSB tax. Future studies should include separate measures of trust in fiscal authorities (e.g., Ministry of Finance, Ghana Revenue Authority).

### Policy, practice, and research implications

The Ministry of Finance should amend the Excise Duty (Amendment) Act 1108 to legally ring-fence at least 50% of sugar-sweetened beverage tax revenues for the National Health Insurance Scheme, specifically for non-communicable disease outpatient services and insulin subsidies. This legislative earmarking would directly address the “tax fatigue” identified in our qualitative theme 3. The Food and Drugs Authority should immediately accelerate the implementation of front-of-pack warning labels, given 81.4% public support. A phased rollout beginning with the most commonly consumed sugar-sweetened beverages (Coca-Cola, FanIce, Chivita) would be feasible. Concurrently, the Ministry of Gender, Children and Social Protection should draft legislative instrument restricting unhealthy food marketing on television and social media between 6:00–20:00 h when children are most exposed.

Longitudinal studies are needed to measure whether stated support translates into sustained acceptance after full implementation. Cost-effectiveness analyses of the full policy bundle versus the SSB tax alone would inform resource allocation. This study directly supports SDG 3 (Good Health and Well-being), specifically target 3.4 (reduce non-communicable disease premature mortality by one-third) and target 3.8 (universal health coverage). It also contributes to SDG 2 (Zero Hunger) by promoting healthier food environments without restricting food access, and SDG 16 (Peace, Justice and Strong Institutions) by emphasizing transparent revenue management and institutional trust (25).

The policy recommendations should be interpreted in light of the cross-sectional design. While public support for FOPWL and FMRP is high, actual implementation may face political and industry resistance not captured in this study. The proposed earmarking of SSB tax revenues for the NHIS requires legislative action and sustained political will, which lies beyond the scope of this research. Future studies should conduct implementation research and political economy analyses to assess feasibility and identify barriers to translating public support into policy action.

### Strengths and limitations

This study has several strengths. First, the use of a convergent mixed-methods design enabled the study to quantify the prevalence and predictors of policy support while also exploring the meanings, concerns, and values underpinning those views. Second, the study examined support for a bundle of complementary food environment policies rather than focusing narrowly on SSB taxation alone. Third, the inclusion of psychosocial constructs such as perceived effectiveness, perceived fairness, and institutional trust provided a more policy-relevant account of support than socio-demographic factors alone.

The findings should be interpreted in light of several limitations. The cross-sectional design does not permit causal inference, and the study was conducted in an urban metropolis, limiting transferability to rural settings. Social desirability bias may have inflated support for health-protective policies. Our measure of political trust was limited to the Ghana Health Service, which does not capture fiscal distrust, a key concern raised in qualitative findings (Theme 3), potentially underestimating the role of revenue management concerns in shaping tax support. Stated support may not translate into sustained acceptance post-implementation.

A further limitation is the use of binary logistic regression (support vs. no support), which discards variance in the 5-point Likert scale. However, this approach was chosen for clinical interpretability and is consistent with prior studies (17). Sensitivity analyses using alternative outcome definitions (support ≥4; support for ≥3 policies) and model specifications (robust standard errors, survey weights) yielded consistent findings. Model diagnostics confirmed adequate fit (VIF mean = 1.38; Hosmer-Lemeshow *p* = 0.40; AUC = 0.81). While these analyses support robustness, future research should employ ordinal logistic regression or latent-variable approaches.

We also did not measure participants’ prior awareness of the Excise Duty Act 1108. According to the Theory of Planned Behavior, prior knowledge is a key determinant of attitude. This omission may introduce omitted variable bias, as some respondents may have reacted to a hypothetical rather than an existing policy. Future studies should assess prior awareness as a control variable.

## Conclusion

In this urban Ghanaian setting, public support for food environment policy extends beyond sugar-sweetened beverage taxation to include front-of-pack warning labeling, restrictions on unhealthy food marketing, and healthier public food procurement. Support was shaped by perceived effectiveness, institutional trust, and perceived fairness. A broader, integrated policy bundle is both socially acceptable and politically feasible, particularly when implementation is transparent and clearly linked to public health benefits. The cross-sectional design prevents causal inference; stated support may not predict real-world acceptance post-implementation. Findings are limited to an urban metropolis and may not generalize to rural Ghana. Social desirability bias may have inflated support for health-protective policies.

### Future research directions

(1) Longitudinal cohort studies tracking policy support before, during, and after implementation; (2) Discrete choice experiments to quantify trade-offs between tax burden and health benefits; and (3) Implementation science research to identify barriers to ring-fencing revenue in Ghana’s fiscal system.

## Data Availability

The raw data supporting the conclusions of this article will be made available by the authors, without undue reservation.
