# Supplementary material for: Beyond the sugar tax: public acceptability of a comprehensive food policy bundle in urban Ghana
Source: Front Nutr. 2026 Aug 25;13:1847802. doi: 10.3389/fnut.2026.1847802 (PMC13549870; doi:10.3389/fnut.2026.1847802)
Supplement: Supplementary file 2 [file Supplementary_File_2.docx]

*Beyond the Sugar Tax: Public Acceptability of a Comprehensive Food Policy Bundle in Urban Ghana*

Kasim Abdulai^1*^, Emil Kafui Amenu^1^, Portia Bangkuu^1^, Ivan Addae-Mensah^1^, Francis Adane^2^, Abass Daudi^3^

^1^Translational Nutrition Research Group, Department of Nutrition and Dietetics, University of Cape Coast, Cape Coast, Ghana

^2^Department of Health Policy, Planning and Management, School of Public Health, University of Ghana, Legon, Accra, Ghana

^3^Tamale Teaching Hospital, Tamale, Ghana

*** Correspondence:**
Corresponding Author
[kasim.abdulai@ucc.edu.gh](mailto:kasim.abdulai@ucc.edu.gh)

**Appendix B: Data Collection Instruments**

**B1. Quantitative Questionnaire**

**SECTION A: Socio-demographic Characteristics**

| **Question** | **Response options** |
| --- | --- |
| What is your sex? | Male / Female / Prefer not to say |
| What is your age group? | 18–29 / 30–39 / 40–49 / 50–59 / 60–69 |
| What is your highest level of education? | No formal education / Primary / Junior High School / Senior High School / Vocational/Technical / Tertiary |
| What is your current marital status? | Single / Married / Divorced / Widowed / Separated |
| What is your main occupation? | Formal employment / Self-employed / Trader / Farmer / Fisher / Unemployed / Retired |
| What is your estimated average monthly income? | <500 GHS / 500–999 GHS / 1,000–1,999 GHS / 2,000–4,999 GHS / ≥5,000 GHS / Prefer not to say |
| How many people live in your household? | 1 / 2–3 / 4–5 / 6–7 / 8+ |
| Do you have any children under 18 years living with you? | Yes / No |
| Do you have any chronic condition (e.g., diabetes, hypertension, heart disease)? | Yes / No / Don't know |
| How often do you consume sugar-sweetened beverages (e.g., Coca-Cola, FanIce, Sprite, Chivita)? | Daily / 2–5 times per week / Once a week / 2–3 times per month / Less than once a month / Never |
| What is your preferred beverage type? | Carbonated soft drinks / Fruit juices / Energy drinks / Sweetened tea/coffee / Water / Other |

**SECTION B: Awareness of Non-Communicable Diseases (10 items)**

*Please indicate if the following statement is* ***True*** *or* ***False****.*

| **Statement** | **True** | **False** |
| --- | --- | --- |
| 1. Eating too much sugar can cause diabetes | ☐ | ☐ |
| 2. High salt intake increases risk of high blood pressure | ☐ | ☐ |
| 3. Lack of fruits and vegetables can lead to heart disease | ☐ | ☐ |
| 4. Physical inactivity is a risk factor for non-communicable diseases | ☐ | ☐ |
| 5. Non-communicable diseases cannot be transmitted from person to person | ☐ | ☐ |
| 6. Obesity increases the risk of developing type 2 diabetes | ☐ | ☐ |
| 7. Smoking tobacco is a major cause of lung cancer and heart disease | ☐ | ☐ |
| 8. Excessive alcohol consumption can cause liver disease and cancer | ☐ | ☐ |
| 9. Regular health check-ups can help detect NCDs early even without symptoms | ☐ | ☐ |
| 10. A family history of diabetes or high blood pressure increases your own risk | ☐ | ☐ |

**SECTION C: Support for Food Environment Policies**

*(5-point Likert scale: 1=Strongly oppose, 2=Oppose, 3=Neutral, 4=Support, 5=Strongly support)*

| **Policy statement** | **1** | **2** | **3** | **4** | **5** |
| --- | --- | --- | --- | --- | --- |
| The government should impose a 20% excise tax on sugar-sweetened beverages (SSB tax) | ☐ | ☐ | ☐ | ☐ | ☐ |
| The government should require front-of-pack warning labels on packaged foods high in sugar, salt, or fat (FOPWL) | ☐ | ☐ | ☐ | ☐ | ☐ |
| The government should restrict advertising of unhealthy foods and beverages to children (FMRP) | ☐ | ☐ | ☐ | ☐ | ☐ |
| The government should ensure that public institutions (hospitals, schools) serve only healthy foods (PFPP) | ☐ | ☐ | ☐ | ☐ | ☐ |

**SECTION D: Perceived Effectiveness, Trust, and Burden**

*(5-point Likert scale: 1=Strongly disagree, 2=Disagree, 3=Neutral, 4=Agree, 5=Strongly agree)*

| **Statement** | **1** | **2** | **3** | **4** | **5** |
| --- | --- | --- | --- | --- | --- |
| These food policies will reduce non-communicable diseases in Ghana | ☐ | ☐ | ☐ | ☐ | ☐ |
| I trust the Ghana Health Service to implement these policies fairly | ☐ | ☐ | ☐ | ☐ | ☐ |
| The SSB tax will place a heavy financial burden on my household | ☐ | ☐ | ☐ | ☐ | ☐ |
| The SSB tax is a fair way to raise revenue for health programs | ☐ | ☐ | ☐ | ☐ | ☐ |
| I believe the government will use SSB tax revenue transparently | ☐ | ☐ | ☐ | ☐ | ☐ |
| Warning labels will help me make healthier food choices | ☐ | ☐ | ☐ | ☐ | ☐ |
| Marketing restrictions will protect children from unhealthy food advertising | ☐ | ☐ | ☐ | ☐ | ☐ |

**SECTION E: Perceived Effectiveness of Policy Bundle (Additional Items)**

*(5-point Likert scale: 1=Strongly disagree to 5=Strongly agree)*

| **Statement** | **1** | **2** | **3** | **4** | **5** |
| --- | --- | --- | --- | --- | --- |
| The SSB tax will reduce sugar consumption in Ghana | ☐ | ☐ | ☐ | ☐ | ☐ |
| FOPWL will help consumers identify unhealthy foods | ☐ | ☐ | ☐ | ☐ | ☐ |
| FMRP will reduce children's exposure to unhealthy food advertising | ☐ | ☐ | ☐ | ☐ | ☐ |
| PFPP will improve the nutritional quality of food in public institutions | ☐ | ☐ | ☐ | ☐ | ☐ |
| The full policy bundle (all four policies together) will be more effective than the SSB tax alone | ☐ | ☐ | ☐ | ☐ | ☐ |

**B2. In-depth Interview Guide (Semi-structured)**

**Introduction:**

*Thank you for participating in this interview. This discussion will take about 30–45 minutes. Your responses are confidential and will not be linked to your identity. We are interested in your honest opinions about government policies related to food and health. There are no right or wrong answers, we want to understand your personal views and experiences.*

**Icebreaker:**

"Please tell us one thing you would like the government to do to help Ghanaians eat healthier."

**Core Questions:**

1. **SSB Tax**
   - What do you think about the government's plan to tax sugary drinks like Coca-Cola and FanIce?
   - *Probe: Is this fair? Why or why not?*
   - *Probe: Do you think this tax will actually reduce sugar consumption?*
   - *Probe: How would this tax affect your household if you buy these drinks regularly?*
2. **Revenue Earmarking**
   - How would you feel if the government used the money from this tax specifically for the National Health Insurance Scheme (NHIS) to treat diabetes and heart disease?
   - *Probe: Would that change your support? Why or why not?*
   - *Probe: What concerns, if any, do you have about how the government manages tax revenue?*
3. **Front-of-Pack Warning Labelling**
   - What is your opinion on requiring food packages to display a warning label if they are high in sugar, salt, or fat?
   - *Probe: Would you find such labels helpful when shopping? Why?*
   - *Probe: Do you think warning labels would influence what you buy or what your family eats?*
   - *Probe: What concerns, if any, do you have about warning labels?*
4. **Food Marketing Restrictions**
   - Do you think the government should ban advertisements for unhealthy foods that target children?
   - *Probe: Why or why not? Can you give an example of an advertisement you have seen?*
   - *Probe: How would such a ban affect children's eating habits in your community?*
5. **Empowerment vs. Coercion**
   - Some people say food taxes are "coercive" (forcing people to change) while warning labels are "empowering" (helping people make informed choices). Do you agree? Please explain.
   - *Probe: Which type of policy do you prefer – informational (labels, marketing bans) or fiscal (taxes)? Why?*
6. **Government's Role**
   - What concerns, if any, do you have about the government telling people what they should or should not eat?
   - *Probe: Is this the government's role? Or should individuals make their own choices?*
   - *Probe: Where do you draw the line between public health protection and personal freedom?*
7. **Closing and Future Aspirations**
   - If you could design one policy to improve eating habits in Ghana, what would it be? Why?
   - *Probe: How would you ensure the policy is accepted by the public?*
   - *Probe: What barriers do you think would need to be overcome?*
   - Is there anything else you would like to add about how the government can help Ghanaians eat healthier?

**Closing:**

*Thank you for your time and honest responses. Your input is very valuable to our research. If you have any further thoughts or questions, please feel free to contact the research team using the contact details on the consent form.*
