# Supplementary material for: Beyond the sugar tax: public acceptability of a comprehensive food policy bundle in urban Ghana
Source: Front Nutr. 2026 Aug 25;13:1847802. doi: 10.3389/fnut.2026.1847802 (PMC13549870; doi:10.3389/fnut.2026.1847802)
Supplement: Supplementary file 3 [file Supplementary_File_1.docx]

**Appendix A: Study Area Map**

***Figure 1. Study Area Map***
